# Methods of Measuring and Processing Signals during Tests of the Exposure of a Motorcycle Driver to Vibration and Noise

**DOI:** 10.3390/ijerph16173145

**Published:** 2019-08-28

**Authors:** Tomasz Figlus, Piotr Szafraniec, Tomáš Skrúcaný

**Affiliations:** 1 Faculty of Transport, Silesian University of Technology, 8 Krasinskiego Street, 40-019 Katowice, Poland; 2 Faculty of Operation and Economics of Transport and Communications, University of Žilina, 1 Univerzitna Street, 010-26 Žilina, Slovakia

**Keywords:** exposure, motorcycle, motorcyclist, noise, vibration, signal processing

## Abstract

The paper presents a new method of measuring a motorcycle driver’s exposure to vibration and noise. This method uses the simultaneous measurement of vibration and noise at the points of their direct influence on the motorcycle driver, and the measurement is carried out in real traffic conditions. The vibration of the motorcycle’s handlebars, footrest and seat as well as the noise reaching the beginning of the motorcyclist’s ear canal are recorded. These signals correspond to the direct impact of vibration energy on the upper and lower limbs as well as the torso of a motorcycle driver and the acoustic energy reaching the motorcyclist’s hearing organs. The paper also proposes a signal processing method which enables determination of vibroacoustic effects on the motorcyclist without frequency correction of signals and with taking into account the current standards, and therefore with the use of appropriate frequency corrections. The measurement section of the paper presents examples of results of measurements of the actual exposure of the motorcycle driver to vibration and noise.

## 1. Introduction

Vibration and noise are among the most negative impacts of means of transport on people [1,2,3] and the environment [4,5,6,7,8].

The sources of vibration present in the means of transport include, inter alia, engines, drive train, suspension, tyre contact with the road pavement as well as wear and clearances [9,10,11,12]. Direct human contact with a vibrating machine can lead to changes in the human body, including long-term illness [1,2,13,14,15]. The cause-and-effect relationship between vibration and illness is often not directly evident, e.g., due to the passage of time [1,2,16]. The exposure of humans and their organs to vibration depends on the area where vibration penetrates into the body [2,14,15]. 

Noise pollution represents a major health problem in modern society, leading to a whole host of health effects if not properly monitored and assessed: sleep disorders with awakenings [17], learning impairment [18,19,20], ischemic heart disease [21,22] and annoyance [23,24,25]. Noise impact of means of transport may have direct effects on the driver and passengers and indirect effects on people [24,26,27] and the environment in their immediate vicinity [28,29,30,31,32]. In order to attenuate it, studies and mitigation have been conducted worldwide in the last decades as action plans [33,34,35] for the main transportation sources of noise affecting the modern human life style: road traffic [36,37,38,39], railway traffic [40,41], airport [42,43] and port noise [44].

Vibroacoustic interactions have a significant impact on assessment of the quality and comfort of means of transport. Research in this area is carried out as part of the development of means of transport [25,45,46,47] and as part of the assessment of their impact on users [8,14,26,34,35,48]. Measurements of the so-called workplace exposure are also carried out in accordance with the guidelines set out in the relevant standards [15,49]. In the latter case, the vibroacoustic effects mainly affect the comfort of life and the condition of the transport infrastructure as well as infrastructure in the vicinity. In this case, the standards and test methods developed for this impact [50] are used and methods for its reduction are defined [39,51,52,53].

Motorcycles belong to a group of means of transport whose negative impact on people and the environment is significant and can take on different formats. The research carried out in this area can be found, among others, in References [6,54,55,56]. The direct impact of noise and vibration is related to the construction of motorcycles and the close proximity of the driver and passenger to the sources of vibration and noise [10,57,58,59,60,61]. As presented in the papers [62,63,64], professional motorcyclists and frequent users of these means of transport are at risk of hearing loss. The studies presented in Reference [65] indicate that motorcycle users are very likely to lose their hearing even when using very noisy motorcycles only once. The problem of hearing loss and/or tinnitus is present in many motorcycle users [6]. The sources of this exposure are both engine and drive train noise, noise related to the aerodynamics of the motorcycle and its occupants, and air turbulence. Tests carried out in Reference [64] showed a much greater hearing loss in police officers using motorcycles on duty than in police officers performing typical office work.

Vibration interactions include primarily the transmission of vibration through the structure of the motorcycle from its sources, i.e., the engine, drive train and tyre-pavement contact, to the place of their reception, i.e., the upper limbs, lower limbs and trunk of the motorcyclist and passenger. The exposure to lower back pain is discussed in Reference [3] and problems with finger and shoulder diseases in References [61,66] The studies on exposure of motorcyclists to general vibration were carried out, among others, in Reference [57]. These studies assessed typical motorcycles used in Taiwan, which are often the main transport vehicle. These studies have shown instances where vibration can be exceeded, adversely affecting the drivers’ health.

Motorcycle handlebar vibration tests carried out in Reference [67] led to the conclusion that prolonged use of a motorcycle may cause significant adverse effects on the drivers’ health. In Reference [13], a high exposure of the health of motorcyclists to both hand-arm vibration and general vibration was demonstrated.

**Figure 1:**
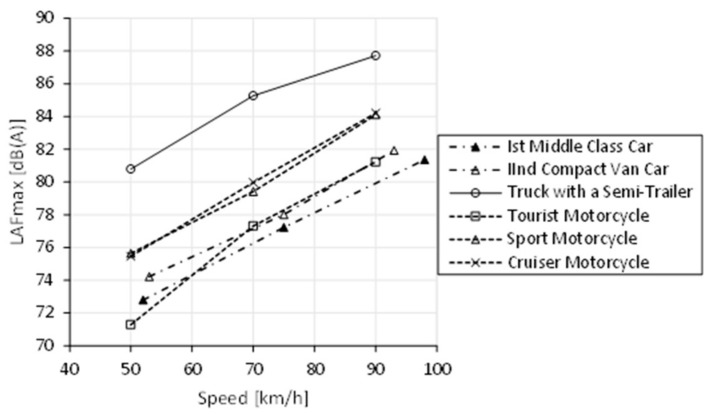
Influence of different means of transport and their travelling speed on the maximum sound level near the roadway (measured at a distance of 7.5 m from the vehicle movement axis) [8].

On the other hand, the vibration impact of motorcycle traffic on the surrounding environment is low, while an important role may be played by noise generated by these means of transport. As the authors’ research presented in Reference [8] shows, the noise level is highly dependent on the type of motorcycle and the exhaust system used [45]. Examples of test results in Figure 1 indicate that the level of noise generated by motorcycles is similar or even higher than noise generated by passenger cars. 

**Figure 2:**
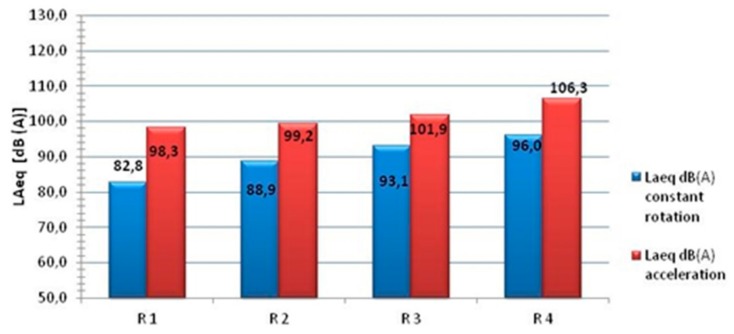
Influence of the type of motorcycle exhaust system on the sound level in the area of the motorcyclist’s head [45].

The use of different types of exhaust systems in motorcycles significantly contributes to the change in the generated sound level [45]. The results of the tests in Figure 2 indicate that the motorcycle noise recorded near the area of the motorcyclist’s head, regardless of the silencer used (R1–R4), was above 80 dB(A). Therefore, if a motorcycle is used for a longer period of time, it can affect the motorcyclist’s hearing.

The use of means of transport also involves the possibility of their involvement in various road situations. The research presented in papers [68,69,70,71] discusses how motorcyclists and other drivers perceive and behave in different situations on the road. The factors, presented in papers [72,73], that describe the sensory and cognitive visibility of motorcycles may also be disturbed by noise and vibration occurring in the means of transport. Noise and vibration to which motorcyclists are exposed can also reduce their concentration and alertness when driving a motorcycle, which can consequently lead to greater risk-taking by the drivers.

The paper presents a new method of measuring and processing signals during tests of a motorcycle driver’s exposure to vibration and noise. The measurement method uses simultaneous measurement of vibration and noise in places where they directly affect the motorcycle driver, and in the signal processing method two ways of determining the effects of vibrations and noise on motorcycle drivers were proposed. The methods were tested during the measurements of the motorcyclist’s exposure to vibration and noise in real traffic conditions, where the basic vibroacoustic effects on the motorcyclist were determined.

## 2. Method of Measuring the Impact of Vibration and Noise on a Motorcyclist

So far, the assessment of the vibration impact has been carried out with the use of devices which, in separate measurements, recorded significant vibration acceleration values in different locations on the motorcycle. The measurement of the noise reaching the motorcycle’s hearing organ when travelling in real conditions—the measurement of the sound level under the helmet in the area of the auricle and the beginning of the external auditory canal—has not yet been analysed in detail.

At present, there are known devices for measuring vibration and noise of machines and devices, as well as their impact on humans, which are used to study the level of vibroactivity and noisiness and to analyse the technical condition and environmental hazards. Local vibration can be measured with single-axis and three-axis vibration acceleration transducers, which allow the vibration level to be determined depending on, for example, the current technical condition of the machine or device, but also, for instance, depending on the condition of the road surface. Noise measurements use microphones with sound analysers which enable, for example, the measurement of the sound level at workstations, and binaural microphones, which enable the measurement of sound directly reaching the human ear and which are used, e.g., to record music. The presented test methods have been described, among others, in PN-EN ISO 5349–1: 2004 [74], PN-EN ISO 5349–2: 2004 [75], PN-EN 14253 + A1: 2011 [76], ISO 2631–1: 1997 [77], PN-N-01307:1994 [78] and PN-EN-ISO 9612: 2011 [79].

As part of the paper a new method of measuring a motorcycle driver’s exposure to vibration and noise was developed [80]. This method uses the simultaneous measurement of vibration and noise at the points of their direct influence on the motorcycle driver. The method was used to measure the motorcyclist’s exposure to vibration and noise in real traffic conditions.

Parallel recording and analysis of vibration and noise using the proposed method enables global assessment of the motorcyclist’s exposure to vibration and noise. By analysing, among others, time and frequency waveforms of the recorded values, it is possible to assess the process of influence of vibration and noise on humans, taking into account the propagation of noise and vibration energy and identification of places where this impact causes the greatest nuisance. Knowledge of this information can help to assess a motorcyclist’s local and global exposure to vibration and noise, and is a valuable source of information on the vibroacoustic characteristics of the area where vibration and noise are transmitted, as well as on the possibilities of designing new solutions with reduced vibration and noise levels.

The method of measurement is based on the fact that, unlike the methods used so far, the actual assessment of the impact of vibration and noise on a motorcyclist is performed by using parallel measurements of vibration affecting the upper and lower limbs of a person and his torso together with internal organs in three mutually perpendicular directions, as well as the measurement of noise reaching the human hearing organ. The advantage of this method of measurement is the fact that on the basis of the recorded vibration and noise signals it is possible to conduct a parallel analysis of the recorded values, obtain characteristics of noise and vibration energy propagation to a person, and assess the level of global exposure of a person to vibration and noise, while taking into account their different propagation paths.

In the developed method, the measuring device used is characterised by the fact that it consists of a set of measuring instruments comprising two three-axis vibration acceleration transducers and a measuring pad equipped with one three-axis transducer, as well as binaural microphones and a data acquisition system. Transducer 1 is attached to the handlebars of the motorcycle, in the handle, in the area immediately adjacent to the position where the motorcyclist’s hand is located. Transducer 2 is attached to the footrest in the area where the motorcyclist’s foot is located. The measuring pad with Transducer 3 is mounted on the motorcycle’s seat, in the place occupied by the motorcyclist during normal use. Binaural Microphones 4 are mounted in the area of the motorcyclist’s auricle at the beginning of the external auditory canal under the helmet. Measuring Transducers 1 and 2, measuring Pad 3 and Microphones 4 are connected to the Data Acquisition System 5, in which the measured values can be recorded or transmitted to the next recording device.

**Figure 3:**
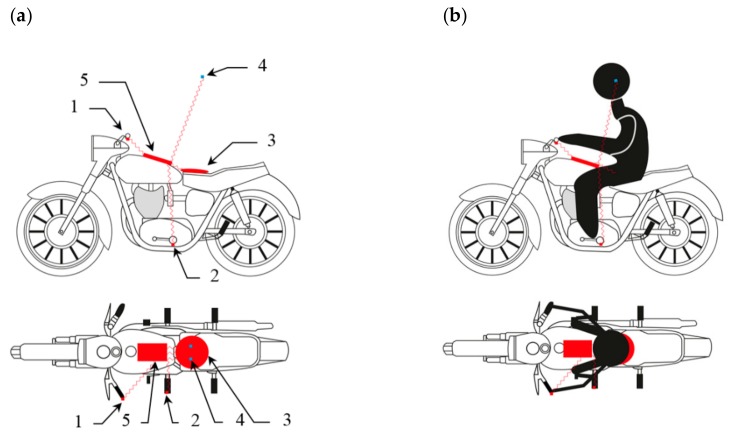
Diagram of the arrangement of the sensors in a system without (**a**) and with the motorcyclist (**b**), where [80]: 1–2—3-axis transducers, 3—measuring pad with a 3-axis transducer, 4—4’-binaural microphones, 5—recording system.

The reference diagram of the mounted device is shown in Figure 3 in a system with and without the motorcycle driver.

The proposed method is an optimal solution for assessing a motorcyclist’s global exposure to vibration and noise, both in real traffic measurements and in laboratory conditions. It is characterised by full information on the characteristics of vibration and noise in areas where they affect people, which in turn makes it possible to obtain a full picture of the vibroacoustic hazards to which a motorcyclist is exposed. This method also makes it possible to eliminate errors resulting from vibration measurements which so far have been carried out separately and which do not take into account exposure to noise among the motorcyclist’s health risks.

## 3. Application of the Developed Measurement Method

The measuring system selected for active experiments is presented in this section on the basis of the theoretical method for measuring vibration and noise affecting a motorcyclist in real traffic conditions, developed and presented in Section 2. Preliminary tests were carried out in order to validate the measuring system for road tests, and then measurements were made on selected motorcycle.

The test experiment was conducted on one tourist motorcycle equipped with an engine with a cylinder capacity of 599 cm^3^, maximum power of 57 kW (76 hp) at 10,500 min^−1^ and torque of 58 Nm at 8000 min^−1^. It was a used motorcycle in a good technical condition.

The motorcycle was driven by a man with a height of 183 cm and a weight of 80 kg. The research was carried out on a straight section of a road with an undamaged asphalt pavement. In the experiments, the following assumptions were made regarding the speed of the motorcycle and the selected gear of the drive train:

* tests at a constant motorcycle travelling speed:

- travelling speed of 50, 70, 90 and 110 km/h,

- the selected gear: 2, 3, 4 and 5 (selecting the gear at low motorcycle travelling speeds was supposed to ensure stable engine operation),

* examinations during the acceleration test:

- change of speed in the 45–110 km/h range,

- selected gear 3—gear selection ensured a wide range of motorcycle engine rotation changes.

The research assumes that the influence of the motorcycle on the driver is a typical influence of a motorcycle on the motorcyclist and therefore does not require any additional ethical approvals.

The measurements were carried out using a measurement system consisting of:

- two 3-axis PSB transducers, model 356A02,

- two OKM II Rock-Klassik Studio Version binaural microphones made by Soundman,

- measuring pad with a Svantek 3-axis transducer, type SV 39,

- LMS Scadas XS recording system from Siemens, which enables simultaneous recording of the motorcycle’s GPS position together with the dedicated measurement control software—installed on a tablet type portable computer.

**Figure 4:**
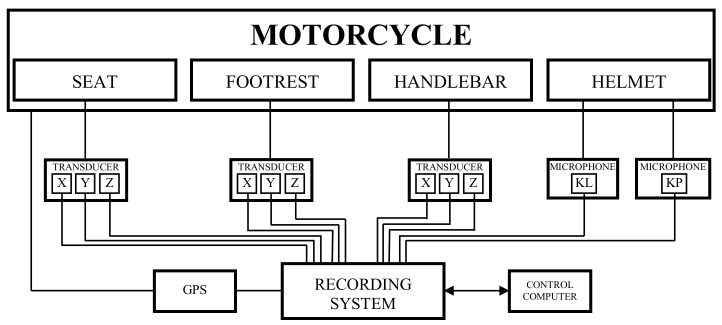
Measuring system diagram.

**Figure 5:**
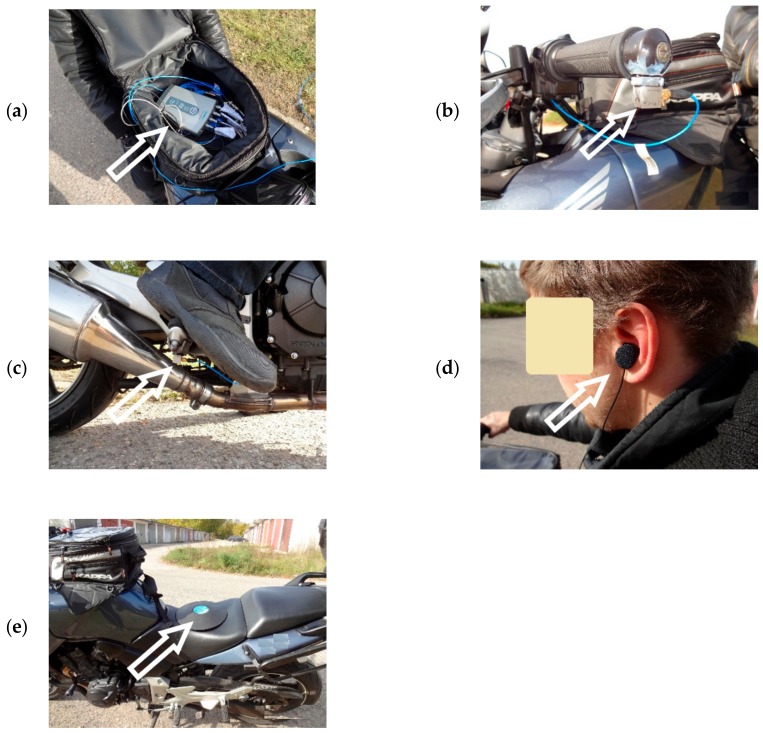
Arrangement of the measuring system’s elements: (**a**) signal recording system, (**b**) three-axis transducer installed on the handlebars of the motorcycle, (**c**) three-axis transducer installed on the footrest of the motorcycle, (**d**) binaural microphone placed in the motorcyclist’s ear, (**e**) measuring pad with a three-axis transducer.

Figure 4 shows the diagram of the measuring system and Figure 5 shows the locations where the measuring system’s elements were installed on the selected measurement motorcycle.

## 4. Signal Processing Method

The developed method enables the analysis of acceleration of vibration recorded in different locations on the motorcycle—points of direct contact between the driver and the motorcycle—and the level of sound pressure present in the motorcycle helmet at the beginning of the driver’s ear canal. Since the impact of vibration and noise on people is one of the constantly recognised methods of research in the field of occupational health and safety, as well as medical research, it has been assumed that the signal processing methods should enable two types of analysis to be carried out. In the former case in the entire frequency band of the recorded signals not previously subjected to corrective filtering, and in the latter case for signals subjected to the frequency correction according to the recommendations of the relevant national and European standards PN-EN ISO 5349-1:2004, PN-EN ISO 5349-2:2004, PN-EN 14253+A1:2011, ISO 2631-1:1997, PN-N-01307:1994 and PN-EN-ISO 9612:2011. See References [81,82,83] for the threshold and limit values of the recorded signals.

**Figure 6:**
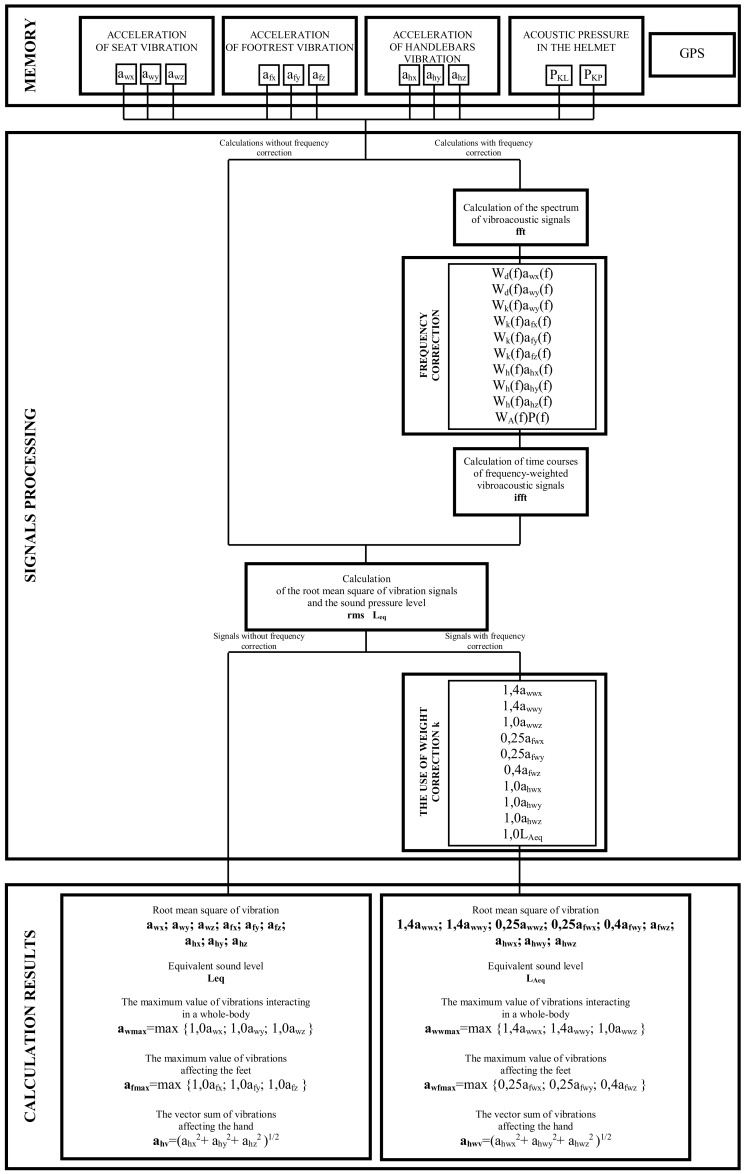
Method of determining signals for analyses.

Figure 6 shows a diagram of the method of determining signals for further analyses. The most important symbols on the diagram:

W_h_, W_d_, W_k_, W_A_—frequency weighing characteristics,

x, y, z—vibration measurement directions,

fft—fast Fourier transform,

rms—root mean square of the signal.

## 5. Results and Discussion

### 5.1. Research in the Time Domain

**Figure 7:**
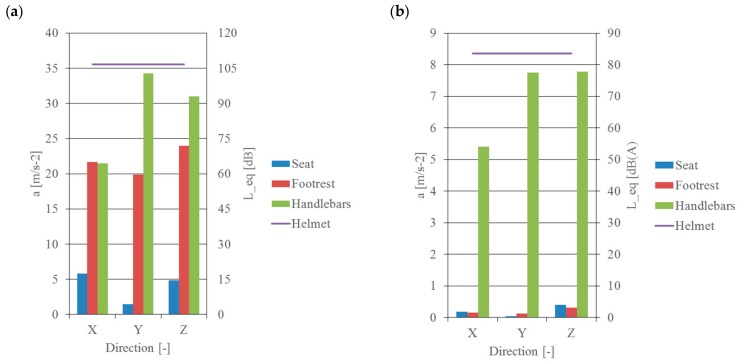
Values of recorded vibration acceleration depending on the direction of measurement and noise: (**a**) signals not frequency corrected, (**b**) frequency corrected signals

The real levels of vibration and noise, which are present during the operation of a motorcycle and to which the motorcyclist is exposed, were analysed on the basis of the signals recorded during road measurements. In these tests, the directional distribution and the effective value of vibration at selected measurement points (their impact on humans), recorded at a constant travelling speed of 50 km/h, were evaluated. The levels of the sound in the helmet recorded at that time were also analysed. The results of these calculations are shown in Figure 7.

These tests show that the greatest human exposure to vibration occurs from the handlebars to the upper limbs, smaller from the footrest of the motorcycle to the lower limbs, and the smallest from the seat to the torso (i.e., exposure to general vibration). The tests were carried out in three mutually perpendicular directions. When analysing the distribution of vibration energy in each of the directions considered, it can be observed that the highest vibration acceleration values were recorded in the Y and Z direction, i.e., perpendicular to the direction of motion. Direction X—parallel to the direction of motion—was characterised by the lowest level of vibration amplitude for interaction from the seat and handlebars. 

In the next stage of the research signals recorded at various quasi-stationary values of motorcycle travelling speeds were analysed. During these measurements, the motorcycle travelled at speeds of 50, 70, 90 and 110 km/h in gear 2, 3, 4 and 5 respectively. The aim of these experiments was to analyse the influence of such factors as travelling speed, engine speed and motion resistance on vibration and noise affecting the motorcyclist. 

**Figure 8:**
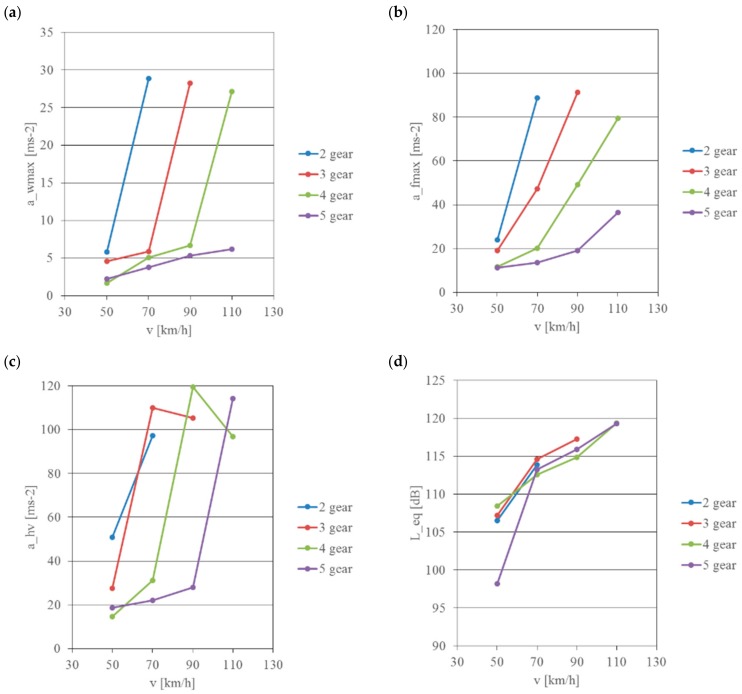
Change of noise and vibration acceleration depending on the travelling speed and the selected gear—signals not frequency corrected: (**a**) measuring point—seat, (**b**) measuring point—footrest, (**c**) measuring point—handlebars, (**d**) measuring point—helmet.

**Figure 9:**
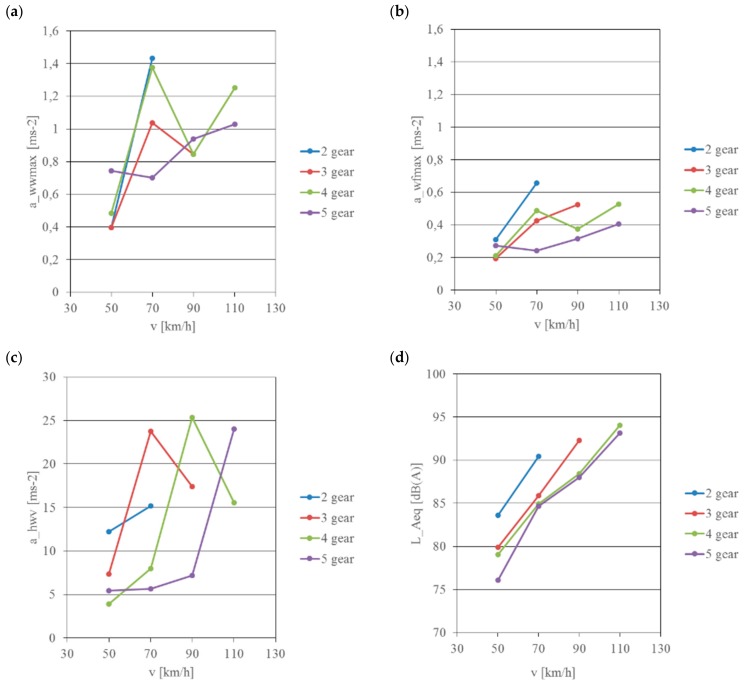
Change of vibration acceleration and noise depending on the travelling speed and the selected gear—frequency corrected signals: (**a**) measuring point—seat, (**b**) measuring point—footrest, (**c**) measuring point—handlebars, (**d**) measuring point—helmet.

Figure 8 and Figure 9 show values calculated without using correction curves as well as frequency corrected values.

The general vibration acting on the driver’s torso is significantly lower than vibration present on the footrest and handlebars. The vibration levels are in the range of 1.7–28.83 m/s^2^ for the seat and 11.2–119.47 m/s^2^ for the footrest and handlebars. With unadjusted measurements of the sound pressure level—linear measurement in the entire band—the value of noise increases non-linearly with the increase in motorcycle travelling speed and ranges from 98.18 dB to nearly 119.35 dB. When analysing the test results, it can be observed that in order to reduce the negative impact of the motorcycle on the driver the highest operationally viable gear ratio of the motorcycle gearbox should be used for propulsion as quickly as possible.

Carrying out vibration acceleration processing using appropriate corrective characteristics W_d_, W_k_ and W_h_ and application of weight coefficients k made it possible to calculate levels of vibration acting on the driver through the seat, footrest and handlebars of the motorcycle, and application of correction A enabled calculation of noise reaching his hearing organ.

The calculated vibration accelerations indicate significant non-linear changes in their values regardless of the motorcycle’s exposure point. The recorded vibration values are in the range of 0.39–1.43 m/s^2^ for the motorcycle seat and 0.21–0.52 m/s^2^ for the footrest. The recorded vibration acceleration values for the seat in most cases exceed the daily mechanical vibration exposure limit values (A_(8)limit_ = 0.8 m/s^2^), but are lower than the short-duration mechanical vibration exposure limit value (a_w,30 min, limit_ = 3.2 m/s^2^).

The values of local hand-arm vibration acceleration, i.e., values recorded on the handlebars, were each time higher than the daily mechanical vibration exposure limit value (A_(8)limit_ = 2.8 m/s^2^) and at a higher engine speed they were higher than the short-duration mechanical vibration exposure limit value (a_w,30min, limit_ = 11.2 m/s^2^). Measurements of the sound level in the helmet using correction curve A indicate that sound level 84 dB(A) is exceeded already at a motorcycle speed of 70 km/h. The recorded maximum values were above 94 dB(A).

### 5.2. Research in the Frequency Domain

The impact of vibration and noise generated by means of transport on users and the environment should also be considered in the frequency aspect. This approach allows identification on the basis of the recorded vibroacoustic signals, their main components and their frequency distributions. The assessment of the phenomenon of generation, propagation and influence of vibration and noise carried out based on this is the basis for conclusions on the impact of these signals on the health and comfort when using means of transport.

**Figure 10:**
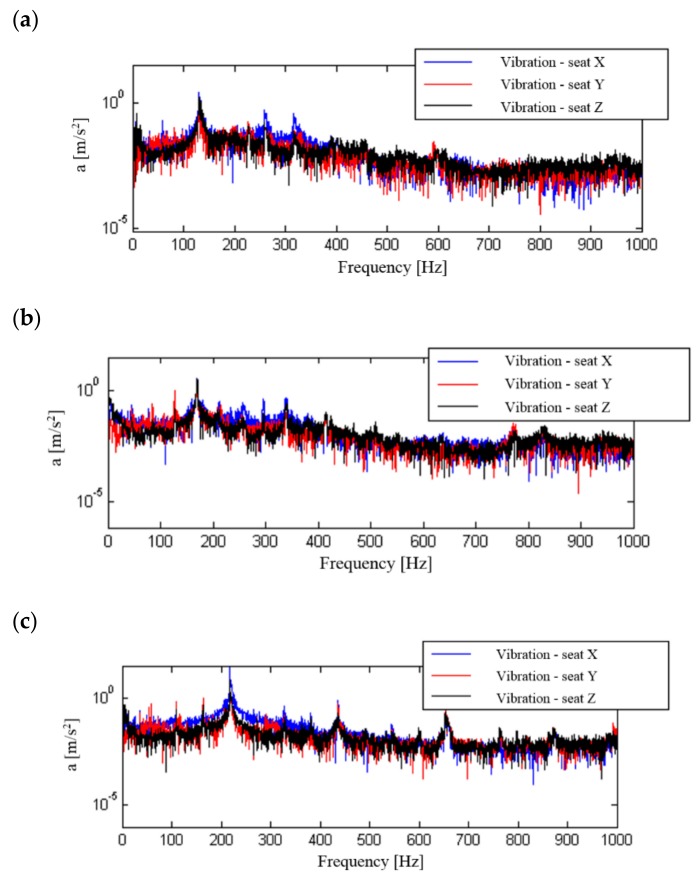
Frequency analysis of general vibration (seat) in the X, Y and Z directions: (**a**) speed 50 km/h, (**b**) speed 70 km/h, (**c**) speed 90 km/h.

**Figure 11:**
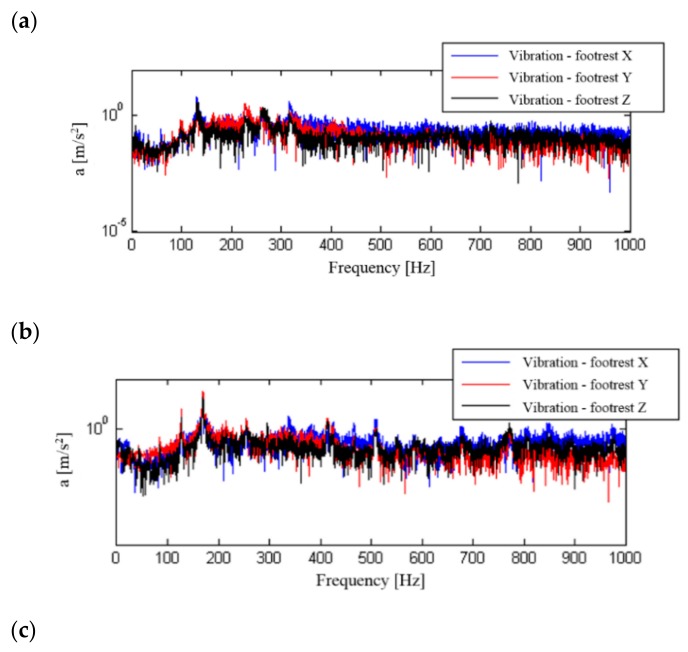

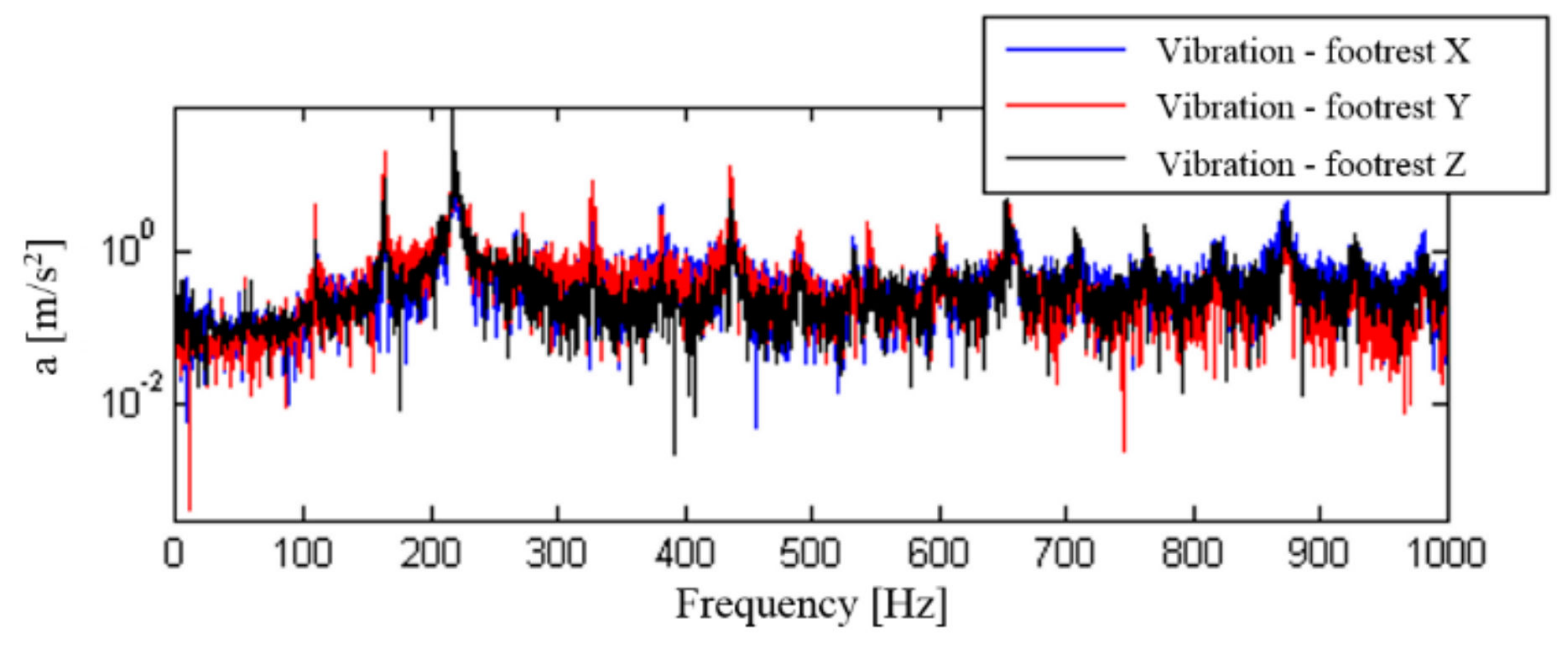
Frequency analysis of general vibration (footrest) in the X, Y and Z directions: (**a**) speed 50 km/h, (**b**) speed 70 km/h, (**c**) speed 90 km/h.

**Figure 12:**
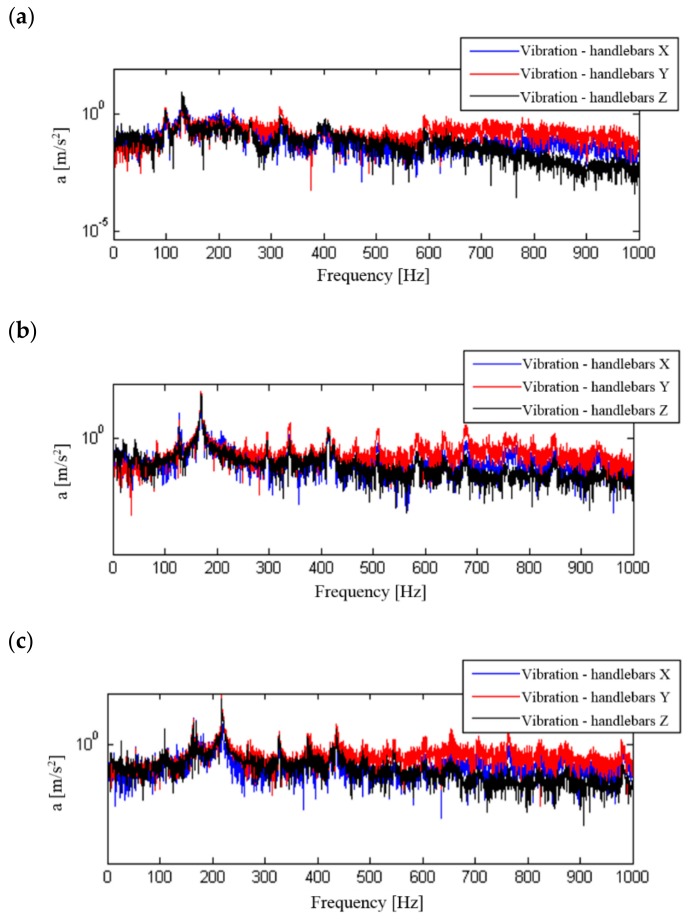
Frequency analysis of general vibration (handlebars) in the X, Y and Z directions: (**a**) speed 50 km/h, (**b**) speed 70 km/h, (**c**) speed 90 km/h.

**Figure 13:**
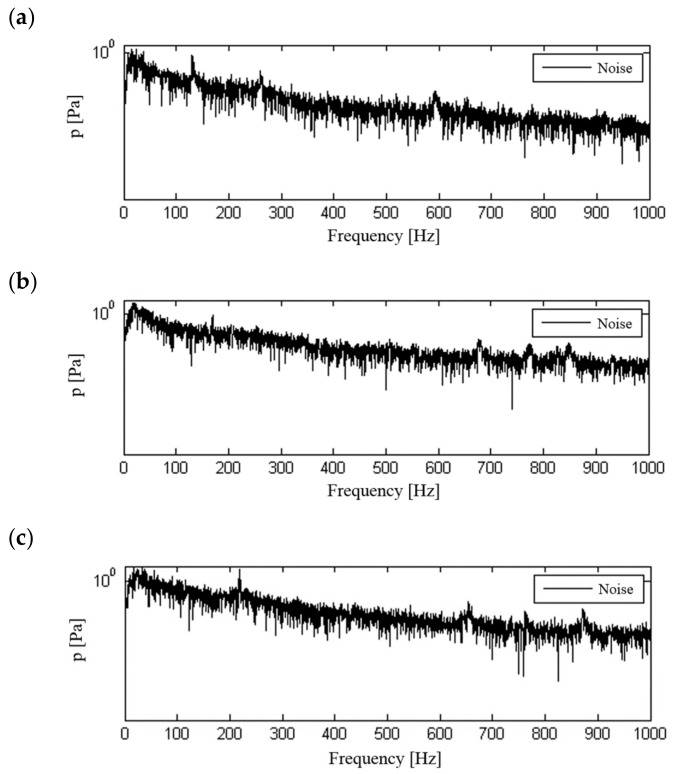
Frequency analysis of sound pressure in the helmet: (**a**) speed 50 km/h, (**b**) speed 70 km/h, (**c**) speed 90 km/h.

A frequency analysis of the influence of the change of motorcycle travelling speed (engine speed) on the structure of signal spectra and their influence on the motorcyclist was carried out. Signal processing was performed for signals that were not frequency corrected. The calculation used signals recorded at a constant motorcycle speed of 50, 70 or 90 km/h in the third gear, which allowed the use of low, medium and high internal combustion engine revolutions. The calculated spectra for the recorded signals are shown in Figure 10, Figure 11, Figure 12 and Figure 13.

The determined signal spectra indicate that the dominant frequencies of vibration and noise signals are those associated with the combustion process in the engine. They occur in the frequency range above 100 Hz and increase with the motorcycle’s speed and therefore with the engine speed. At a travelling speed of 70 km/h and 90 km/h, in the spectrum of signals recorded for the seat and footrest of the motorcycle, there are additional frequencies that are significantly lower than the dominant frequency. When analysing the noise spectrum, in addition to the combustion frequency in the engine, an increase in noise can also be observed at increasing travelling speeds in the low frequency ranges.

In further studies, the change in the structure of the spectra of the recorded vibration and noise was evaluated. This test type makes it possible to examine the main components present in the signals and their impact on motorcyclists at a variable motorcycle travelling speed (motorcycle engine speed). Time and frequency distributions of signals recorded during the acceleration of the motorcycle were calculated. Short-duration spectra were calculated using a fast Fourier transform (fft). The signals recorded during the rapid acceleration of the motorcycle from the speed of about 45 km/h to about 100 km/h in the third gear, frequency corrected and not, were used for the calculations.

**Figure 14:**
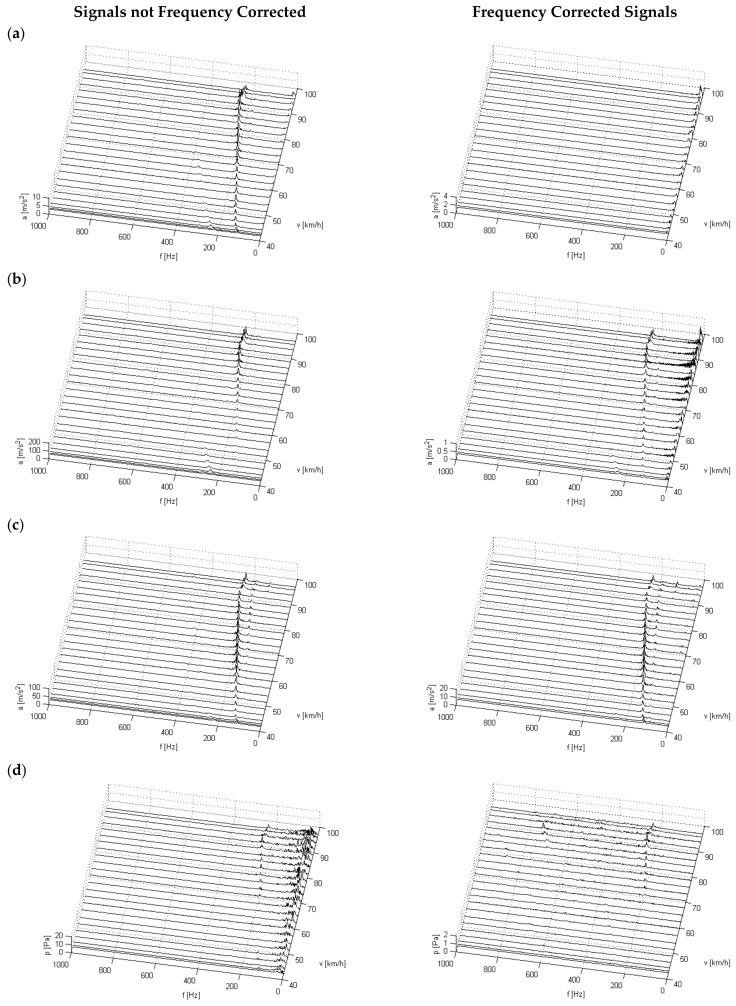
Time-frequency distributions of acceleration of vibration in the Z direction and noise—frequency corrected and not: (**a**) measuring point—seat, (**b**) measuring point—footrest, (**c**) measuring point—handlebars, (**d**) measuring point—helmet.

Figure 14 shows the calculated time and frequency distributions of vibration affecting the driver through the seat and lower and upper limbs in the Z direction, as well as the recorded sound pressure level in the helmet.

Time and frequency distributions of vibration enable identification of changes in spectrum components depending on the motorcycle travelling speed and, therefore, its engine speed. In these distributions, a significant increase in signal amplitudes can be observed due to the combustion process and its harmonics in the range from about 100 Hz to about 250 Hz. The bands of these frequencies are predominant in the spectrum regardless of the measuring point and overlap in the selected speed ranges with the natural vibration of the selected organs of the human body.

The change of the spectrum structure in the low-frequency range—up to 80 Hz—is characterised by low energy values of vibration levels. As the speed increases, an increase in amplitude of these frequencies is also observed, but their value is significantly lower than the frequency of the combustion process in the engine. The signal processing carried out with the use of band-pass filters made it possible to analyse the exposure to vibration transmitted by the human body with general and local effects. In this case, increases in the amplitude of low-frequency vibration, and therefore an increase in exposure of humans to these effects, was observed. It can be noted, however, that the application of these filters leaves in the vibration signal a strongly energetic component associated with the combustion process.

Measurements and processing of noise signals not subjected to frequency filtration tests indicate strongly energetic changes in the amplitude of sound pressure in the low-frequency range. The amplitude value increases as the rotational speed increases. When using correction Filter A, this noise becomes significantly lower energetically in the low-frequency range and therefore, despite the increase in its amplitude, it does not create a significant nuisance for the motorcycle driver. However, local increases in sound amplitude can be observed in this signal at a higher engine speed, which is caused by the fact that filtration A leaves the components associated with the combustion process, i.e., frequencies above 200 Hz.

## 6. Conclusions

At present, means of road transport are subject to increasingly higher requirements in terms of minimising their negative impact on the environment and ensuring high comfort of use. These requirements mean that the development of means of transport requires the use of highly advanced design methods and complex systems for testing prototypes.

The emission of vibration and noise is among the significant impacts of means of transport on the environment and their users. In vehicles, this problem can be considered as an aspect of ensuring a sufficiently high level of comfort of use, and also as a factor contributing to health deterioration. While analysing the literature, it was found that motorcycles belong to the group of means of transport which have a negative impact on their users. The main problem here is the exposure of motorcyclists to vibroacoustic signals penetrating the human body through the upper limbs, lower limbs, trunk and hearing organs.

The method of measurement of vibroacoustic signals proposed in the paper, which takes into consideration general vibration, vibration affecting lower and upper limbs as well as noise present in the helmet, is a new approach to the possibility of assessing vibroacoustic effects on motorcyclists. It also allows a broader look on the motorcycle as an object that generates vibration and noise. The use of the measurement system proposed in the publication, which carries out the measurement simultaneously in various areas of the motorcyclist’s exposure, enables a later analysis of specific cases of interactions. This type of measurement system also enables analyses with high sampling rates, which is particularly important when analysing fast changing phenomena. In this case, traditional signal analysers, such as those that determine the equivalent sound level or the maximum sound level, are not sufficient.

In the paper this method was used to evaluate the exposure of motorcyclists to vibration and noise and to compare the level of this exposure using different types of motorcycles. The developed method can also be used to evaluate the motorcycle itself as a source of vibroacoustic signals, the reduction of which is one of the most important tasks pursued in research and development centres dealing with the comfort of this type of means of transport. 

The conducted identification tests of the proposed method of measurements in real traffic made it possible to record the waveforms of vibration signals of selected elements of the motorcycle and noise present in the motorcyclist’s helmet. This therefore confirmed the practical utility of the method for testing motorcycles and their vibroacoustic impact on users in real traffic, as proposed in the patent application.

In connection with the information contained in numerous scientific papers on possible negative impacts of vibration and noise on humans in a wider frequency range than previously considered in the standards, the paper analyses signals in the entire frequency range of signals not subjected previously to the recommended corrections, and applies recommendations of standards for the relevant interactions. These studies show that vibration and noise not subjected to frequency correction were characterised by significantly higher values than frequency corrected signals. Consequently, significant vibroacoustic effects on motorcyclists occur in frequency bands which are omitted from the standards or which are in a near range of frequency bands recommended by them.

When comparing the test results, it can be concluded that the level of vibration affecting the driver through the motorcycle’s seat is the lowest among those recorded. Vibration affecting the lower and upper limbs is significantly higher than for the seat. When analysing the vibration and noise values, it can also be concluded that the vibration levels of the seat and footrest do not exceed the limit values currently adopted in the standards, while the handlebar vibration and the sound level in the helmet may exceed these values. This depends mainly on the way the motorcycle is used and on its type.

## References

[1] Griffin M.J. (2007). Discomfort from feeling vehicle vibration. Veh. Syst. Dyn..

[2] Griffin M.J. (1990). Handbook of Human Vibration.

[3] Palmer K.T., Coggon D.N., Bendall H.E., Pannett B., Griffin M.J., Haward B. (1999). Whole-body Vibration: Occupational Exposures and Their Health Effects in Great Britain. Health and Safety Executive Contract Research Report.

[4] Faberi M., Martuzzi M., Pirrami F. (2004). Assessing the Health Impact and Social Costs of Mopeds: Feasibility Study in Rome.

[5] Makarewicz R., Kokowski P. (2007). Prediction of noise changes due to traffic speed control. J. Acoust. Soc. Am..

[6] Sandberg U. (2002). Noise Emission from Powered Two-Wheeled Vehicles—Position Paper.

[7] Vogiatzis K.E. (2011). Strategic Environmental Noise Mapping & Action Plans in Athens Ring Road (ATIIKI ODOS)—GREECE. WSEAS Transtactions Environ. Dev..

[8] Figlus T., Gnap J., Skrúcaný T., Szafraniec P. (2017). Analysis of the influence of different means of transport on the level of traffic noise. Sci. J. Sil. Univ. Technol. Ser. Transp..

[9] Drozdziel P., Rybicka I., Madlenak R., Andrusiuk A., Siluch D. (2017). The engine set damage assessment in the public transport vehicles. Adv. Sci. Technol.-Res. J..

[10] Figlus T. (2015). The application of a continuous wavelet transform for diagnosing damage to the timing chain tensioner in a motorcycle engine. J. Vibroeng..

[11] Sága M., Vaško M., Handrik M., Kopas P. (2019). Contribution to random vibration numerical simulation and optimisation of nonlinear mechanical systems. Sci. J. Sil. Univ. Technol. Ser. Transp..

[12] Gaska D., Margielewicz J., Haniszewski T., Matyja T., Konieczny L., Chróst P. (2015). Numerical identification of the overhead travelling crane’s dynamic factor caused by lifting the load off the ground. J. Meas. Eng..

[13] Kowalski P., Zając J. (2013). Risk of mechanical vibrations of users of two-wheeled vehicles—Own research results. Bezpieczeństwo Pr..

[14] Paddan G.S., Griffin M.J. (2002). Evaluation of whole-body vibration in vehicles. J. Sound Vib..

[15] Zhao X., Schindler C. (2014). Evaluation of whole-body vibration exposure experienced by operators of a compact wheel loader according to ISO 2631-1:1997 and ISO 2631-5:2004. Int. J. Ind. Ergon..

[16] Wolfgang R., Burgess-Limerick R. (2014). Whole-body vibration exposure of haul truck drivers at a surface coal mine. Appl. Ergon..

[17] Muzet A. (2007). Environmental noise, sleep and health. Sleep Med. Rev..

[18] Zacarías F.F., Molina R.H., Ancela J.L.C., López S.L., Ojembarrena A.A. (2013). Noise exposure in preterm infants treated with respiratory support using neonatal helmets. Acta Acust. United Acust..

[19] Chetoni M., Ascari E., Bianco F., Fredianelli L., Licitra G., Cori L. (2016). Global noise score indicator for classroom evaluation of acoustic performances in LIFE GIOCONDA project. Noise Mapp..

[20] Minichilli F., Gorini F., Ascari E., Bianchi F., Coi A., Fredianelli L., Licitra G., Manzoli F., Mezzasalma L., Cori L. (2018). Annoyance judgment and measurements of environmental noise: A focus on Italian secondary schools. Int. J. Environ. Res. Public Health.

[21] Dratva J., Phuleria H.C., Foraster M., Gaspoz J.M., Keidel D., Künzli N., Liu L.J., Pons M., Zemp E., Gerbase M.W. (2012). Transportation noise and blood pressure in a population-based sample of adults. Environ. Health Perspect..

[22] Babisch W., Beule B., Schust M., Kersten N., Ising H. (2005). Traffic noise and risk of myocardial infarction. Epidemiology.

[23] Miedema H.M.E., Oudshoorn C.G.M. (2001). Annoyance from transportation noise: Relationships with exposure metrics DNL and DENL and their confidence intervals. Env. Health Perspect.

[24] Michta A., Haniszewski T. (2018). Traffic noise experienced on buses, trams and cars in the urban agglomeration of the city of Katowice. Sci. J. Sil. Univ. Technol. Ser. Transp..

[25] Skrucany T., Kendra M., Skorupa M., Grencik J., Figlus T. (2017). Comparison of chosen environmental aspects in individual road transport and railway passenger transport. Procedia Eng..

[26] Engel Z. (2001). Environmental Protection Against Vibration and Noise.

[27] WHO (1997). Prevention of Noise-Induced Hearing Loss.

[28] Noise Legislation. http://www.epa.vic.gov.au/about-us/legislation/noise-legislation#noiseregs.

[29] Ross B.M. (2001). Noise from traffic as a worldwide policy problem. Noise Control Eng. J..

[30] Sendek-Matysiak E. (2019). Multi-criteria analysis and expert assessment of vehicles with different drive types regarding their functionality and environmental impact. Sci. J. Sil. Univ. Technol. Ser. Transp..

[31] Skrucany T., Sarkan B., Figlus T., Synak F., Vrabel J. (2017). Measuring of noise emitted by moving vehicles. MATEC Web Conf..

[32] Berglund B., Lindvall T., Schwela D., WHO (1999). Guidelines for Community Noise.

[33] Licitra G., Ascari F., Fredianelli L. (2017). Prioritizing Process in Action Plans: A Review of Approaches. Curr. Pollut. Rep..

[34] Rybicka I., Drozdziel P., Stopka O., L’uptak V. (2018). Methodology to propose a regional transport organization within specific integrated transport system: A case study. Transp. Probl..

[35] Poliak M., Poliakova A., Mrnikova M., Simurkova P., Jaskiewicz M., Jurecki R. (2017). The Competitiveness of Public Transport. J. Compet..

[36] Cueto J.L., Petrovici A.M., Hernández R., Fernández F. (2017). Analysis of the Impact of Bus Signal Priority on Urban Noise. Acta Acust. United Acust..

[37] Morley D.W., de Hoogh K., Fecht D., Fabbri F., Bell M., Goodman P.S., Elliott P., Hodgson S., Hansell A.L., Gulliver J. (2015). International scale implementation of the CNOSSOS-EU road traffic noise prediction model for epidemiological studies. Environ. Pollut..

[38] Ruiz-Padillo A., Ruiz D.P., Torija A.J., Ramos-Ridao A. (2018). Selection of suitable alternatives to reduce the environmental impact of road traffic noise using a fuzzy multi-criteria decision model. Environ. Impact Assess. Rev..

[39] Yamamoto K. (2010). Special issue on road traffic noise prediction methods. Acoust. Sci. Technol..

[40] Licitra G., Fredianelli L., Petri D., Vigotti M.A. (2016). Annoyance evaluation due to overall railway noise and vibration in Pisa urban areas. Sci. Total Environ..

[41] Bunn F., Henrique P., Zannin T. (2016). Assessment of railway noise in an urban setting. Appl. Acoust..

[42] Flores R., Asensio C., Gagliardi P., Licitra G. (2019). Study of the correction factors for aircraft noise façade measurements. Appl. Acoust..

[43] Iglesias-Merchan C., Diaz-Balteiro L., Soliño M. (2015). Transportation planning and quiet natural areas preservation: Aircraft overflights noise assessment in a National Park. Transp. Res. Part D Transp. Environ..

[44] Bernardini M., Fredianelli L., Fidecaro F., Gagliardi P., Nastasi M.L., Licitra G. (2019). Noise Assessment of Small Vessels for Action Planning in Canal Cities. Environments.

[45] Figlus T., Wilk A., Liscak S., Kalafarski M. (2013). The influence of muffler type of the exhaust system in the sports motorcycle on the level of the emitted noise. Acta Tech. Corviniensis Bull. Eng..

[46] Sejkorova M., Sarkan B., Madlenak R., Caban J., Marczuk A., Verner J., Hyrslova J. (2018). Effect of ferrocene addition to a gas oil on smoke opacity and engine noise. Przem. Chem..

[47] Šarkan B., Skrúcaný T., Semanová Š., Madleňák R., Kuranc A., Sejkorová M., Caban J. (2018). Vehicle coast-down method as a tool for calculating total resistance for the purposes of typeapproval fuel consumption. Sci. J. Sil. Univ. Technol. Ser. Transp..

[48] Makarewicz R., Kokowski P., Golebiewski R., Galuszka M. (2015). Transportation noise composed of identifiable noise events. Noise Control Eng. J..

[B49-ijerph-16-03145] 49.Regulation of the Minister of Transport no 51 from 22 August 2013.

[B50-ijerph-16-03145] 50.*Regulation (EU)* No 540/2014; dated 16 April 2014.

[51] Department of Transport and the Welsh Office (1988). Calculation of Road Traffic Noise.

[52] (2003). FHWA Traffic Noise Model (FHWA TNM).

[53] Sooriyaarachchi R.T., Sonnadara D.U.J. Development of a Road Traffic Noise Prediction Model. Proceedings of the Technical Sessions Institute of Physics.

[54] Berglund B., Nilsson M.E. Total Annoyance and Perceptually Discernible Noise Sources. Proceedings of InterNoise.

[55] (1998). A New Deal for Transport: Better for Everyone.

[56] Skanberg A., Ohrstrom E. (2002). Adverse health effects in relation to urban residential soundscapes. J. Sound Vib..

[57] Chen H.-C., Chen W.-C., Liu Y.-P., Chen C.-Y., Pan Y.-T. (2009). Whole-body vibration exposure experienced by motorcycle riders—An evaluation according to ISO 2631-1 and ISO 2631-5 standards. Int. J. Ind. Ergon..

[58] Kennedy J., Carley M., Walker I., Holt N. (2013). On-road and wind-tunnel measurement of motorcycle helmet noise. Acoust. Soc. Am..

[59] Lower M.C., Hurst D.W., Thomas A. (1996). Noise levels and noise reduction under motorcycle helmets. Proc.-Inst. Acoust..

[60] Malerba M., Conti P. (2017). Influence of the rider seating position on motorcycle aerodynamic performance. Int. J. Eng. Sci. Innov. Technol..

[61] Ochiai A., Naya Y., Soh J., Ishida Y., Ushijima S., Mizutani Y., Kawauchi A., Miki T. (2006). Do motorcyclists have erectile dysfunction? A preliminary study. Int. J. Impot. Res..

[62] Aldman B., Gustaffson H., Nygren A., Wersall J. (1983). Hearing and motorcycle helmets. J. Traffic Med..

[63] Henderson R. (1975). Effect of Safety Helmets on Auditory Capability.

[64] Lesage F.-X., Jovenin N., Deschamps F., Vincent S. (2009). Noise-induced hearing loss in French police officers. Occup. Med..

[65] Carlsson I. (2002). Personal Communication with Statistician.

[66] Structures of the Hand. https://teachmeanatomy.info/upper-limb/misc/structures-hand.

[67] Noh J.M., Rezali K.A.M., As’arry A., Jalil N.A.A. (2017). Transmission of Vibration from Motorcycle Handlebar to the Hand. J. Soc. Automot. Eng. Malays..

[68] Shahar A., Poulter D., Clarke D., Crundall D. (2010). Motorcyclists’ and car drivers’ responses to hazards. Transp. Res. Part F Traffic Psychol. Behav..

[69] Micucci A., Mantecchini L., Sangermano M. (2019). Analysis of the Relationship between Turning Signal Detection and Motorcycle Driver’s Characteristics on Urban Roads; A Case Study. Sensors.

[70] Motorcycle Accident Cause Factors and Identification of Countermeasures. Volume 2: Appendix/Supplemental Data. https://rosap.ntl.bts.gov/view/dot/5652.

[71] Jelalian E., Alday S., Spirito A., Rasile D., Nobile C. (2000). Adolescent motor vehicle crashes: The relationship between behavioral factors and self-reported injury. J. Adolesc. Health.

[72] Hancock P.A., Wulf G., Thom D., Fassnacht P. (1990). Driver workload during differing driving maneuvers. Accid. Anal. Prev..

[73] Wertheim A.H. (2010). Visual conspicuity: A new simple standard, its reliability, validity and applicability. Ergonomics.

[B74-ijerph-16-03145] 74.PN-EN ISO 5349-1:2004. *Mechanical Vibration—Measurement and Evaluation of Human Exposure to Hand-Transmitted Vibration—Part 1: General Requirements (ISO 5349-1:2001)*; 2004-07-09, Warszawa.

[B75-ijerph-16-03145] 75.PN-EN ISO 5349-2:2004. *Mechanical Vibration—Measurement and Evaluation of Human Exposure to Hand-Transmitted Vibration—Part 2: Practical Guidance for Measurement at the Workplace (ISO 5349-2:2001)*; 2004-07-20, Warszawa.

[B76-ijerph-16-03145] 76.PN-EN 14253+A1:2011. *Mechanical Vibration—Measurement and Calculation of Occupational Exposure to Whole-Body Vibration with Reference to Health—Practical Guidance*; 2011-01-05, Warszawa.

[B77-ijerph-16-03145] 77.ISO 2631-1:1997. *Mechanical Vibration and Shock—Evaluation of Human Exposure to Whole-Body Vibration—Part 1: General Requirements*; 1977-07-15, Geneve.

[B78-ijerph-16-03145] 78.PN-N-01307:1994. *Noise—Permissible Values of Noise in the Workplace—Requirements Relating to Measurements*; 1994-12-30, Warszawa.

[B79-ijerph-16-03145] 79.PN-EN-ISO 9612:2011. *Acoustics—Determination of Occupational Noise Exposure—Engineering Method (ISO 9612:2009)*; 2011-08-11, Warszawa.

[B80-ijerph-16-03145] 80.Figlus, T.; Szafraniec, P.; Żuradzki, K.; Skrucany, T. *Method for measuring vibrations and noise in motorcycle, involves connecting binaural microphones to data acquisition system, connecting ear mantle to external auditory duct, and connecting helmet to data acquisition system*. PL419787-A1, 13 December 2016.

[B81-ijerph-16-03145] 81.The Regulation of the Minister of Labour and Social Policy. *The highest acceptable concentrations and intensities of factors harmful to health in the working environment*. The Journal of Laws No. 217, item 1833, 29 November 2002.

[B82-ijerph-16-03145] 82.The Regulation of the Minister of Economy and Labour. *Amending the regulation on the highest acceptable concentrations and intensities of factors harmful to health in the working environment*. The Journal of Laws, no. 212, item. 1769, 10 October 2005.

[B83-ijerph-16-03145] 83.The Regulation of the Minister of Economy and Labour. *Occupational health and safety during works with exposure to noise or mechanical vibrations*. The Journal of Laws of, No. 157, item 118, 5 August 2005.

